# Baseline Inflammatory Biomarkers Identify Subgroups of HIV-Infected African Children With Differing Responses to Antiretroviral Therapy

**DOI:** 10.1093/infdis/jiw148

**Published:** 2016-05-18

**Authors:** Andrew J. Prendergast, Alexander J. Szubert, Chipo Berejena, Godfrey Pimundu, Pietro Pala, Annie Shonhai, Victor Musiime, Mutsa Bwakura-Dangarembizi, Hannah Poulsom, Patricia Hunter, Philippa Musoke, Macklyn Kihembo, Paula Munderi, Diana M. Gibb, Moira Spyer, A. Sarah Walker, Nigel Klein

**Affiliations:** 1 Queen Mary University of London; 2 MRC Clinical Trials Unit at University College London; 3 Institute of Child Health, London, United Kingdom; 4 University of Zimbabwe, Harare; 5 Joint Clinical Research Centre; 6 Makerere University College of Health Sciences; 7 Paediatric Infectious Diseases Clinic/Baylor-Uganda, Kampala; 8 MRC/UVRI Uganda Research Unit on AIDS, Entebbe, Uganda

**Keywords:** HIV, Africa, children, inflammation, immunosuppression

## Abstract

***Background.*** Identifying determinants of morbidity and mortality may help target future interventions for human immunodeficiency virus (HIV)–infected children.

***Methods.*** CD4^+^ T-cell count, HIV viral load, and levels of biomarkers (C-reactive protein, tumor necrosis factor α [TNF-α], interleukin 6 [IL-6], and soluble CD14) and interleukin 7 were measured at antiretroviral therapy (ART) initiation in the ARROW trial (case-cohort design). Cases were individuals who died, had new or recurrent World Health Organization clinical stage 4 events, or had poor immunological response to ART.

***Results.*** There were 115 cases (54 died, 45 had World Health Organization clinical stage 4 events, and 49 had poor immunological response) and 485 controls. Before ART initiation, the median ages of cases and controls were 8.2 years (interquartile range [IQR], 4.4–11.4 years) and 5.8 years (IQR, 2.3–9.3 years), respectively, and the median percentages of lymphocytes expressing CD4 were 4% (IQR, 1%–9%) and 13% (IQR, 8%–18%), respectively. In multivariable logistic regression, cases had lower age-associated CD4^+^ T-cell count ratio (calculated as the ratio of the subject's CD4^+^ T-cell count to the count expected in healthy individuals of the same age; *P* < .0001) and higher IL-6 level (*P* = .002) than controls. Clustering biomarkers and age-associated CD4^+^ and CD8^+^ T-cell count ratios identified 4 groups of children. Group 1 had the highest frequency of cases (41% cases; 16% died) and profound immunosuppression; group 2 had similar mortality (23% cases; 15% died), but children were younger, with less profound immunosuppression and high levels of inflammatory biomarkers and malnutrition; group 3 comprised young children with moderate immunosuppression, high TNF-α levels, and high age-associated CD8^+^ T-cell count ratios but lower frequencies of events (12% cases; 7% died); and group 4 comprised older children with low inflammatory biomarker levels, lower HIV viral loads, and good clinical outcomes (11% cases; 5% died).

***Conclusions.*** While immunosuppression is the major determinant of poor outcomes during ART, baseline inflammation is an additional important factor, identifying a subgroup of young children with similar mortality. Antiinflammatory interventions may help improve outcomes.

Approximately 760 000 children are currently receiving antiretroviral therapy (ART), leading to a 40% reduction in human immunodeficiency virus (HIV) infection–related mortality since 2005 [1], despite pediatric ART coverage remaining at only 30% in 2012 [2]. Over 90% of HIV-infected children live in sub-Saharan Africa, where advanced disease and undernutrition contribute to high mortality (3%–19%) in the first year of ART [3–8].

HIV infection is characterized by immune activation in adults, in whom baseline markers of inflammation and coagulation predict mortality independently of CD4^+^ T-cell count and HIV viral load [9–17]. Much less is known about the causes and consequences of inflammation in HIV-infected children [18], and no studies to date have explored associations between baseline levels of inflammatory biomarkers and mortality in children starting ART. Identifying factors associated with morbidity, mortality, and poor immunological response at ART initiation is important, to target future interventions in children who will need to receive lifelong treatment. We therefore characterized immunodeficiency and inflammation in a large cohort of children aged 3 months to 17 years who were starting ART in Uganda and Zimbabwe, to determine the impact of these factors on morbidity, mortality, and immune reconstitution.

## METHODS

In the ARROW trial (ISRCTN24791884), previously untreated Ugandan/Zimbabwean children aged 3 months to 17 years who were eligible for ART on the basis of 2006 World Health Organization (WHO) criteria were randomized 1:1 to undergo clinically driven monitoring versus laboratory plus clinical monitoring for toxicity (via hematological and biochemical analyses) and efficacy (via CD4^+^ T-cell data) [19]. Children were also randomized 1:1:1 in a factorial design to receive lamivudine, abacavir, and nonnucleoside reverse transcriptase inhibitor (NNRTI) continuously (arm A) or induction-maintenance therapy with lamivudine, abacavir, NNRTI, and zidovudine for 36 weeks, followed by lamivudine, abacavir, and NNRTI (arm B) or lamivudine, abacavir, and zidovudine (arm C), as previously described [19]. The NNRTI (nevirapine/efavirenz) was chosen by clinicians. Caregivers gave written consent; older children (age, 8–17 years) aware of their HIV status also gave assent or consent, as per local guidelines. The trial and immunology substudy were approved by Research Ethics Committees in Uganda, Zimbabwe, and the United Kingdom.

### Study Subjects and Measurements

This study used a case-cohort design. Cases were individuals who died, who had new or recurrent WHO clinical stage 4 events during follow-up (to trial closure; median follow-up duration, 4 years), or poor immunological response to ART (percentage of lymphocytes expressing CD4, ≤ 15% through 3 years of first-line ART, allowing a single measurement of >15%). Controls comprised 316 children in a longitudinal immunology substudy (the last 6 months of recruitment [May–November 2008]) and a random 23% sample of all remaining nonsubstudy children, to reach a sample size of 600 children (Supplementary Figure 1).

In cryopreserved plasma samples obtained before ART initiation (ie, at enrollment) or trial screening (maximum, 30 days before enrollment), baseline levels of inflammatory biomarkers (C-reactive protein [CRP], tumor necrosis factor α [TNF-α], interleukin 6 [IL-6], and soluble CD14 [sCD14]) and interleukin 7 (IL-7) were measured by an enzyme-linked immunosorbent assay (R&D Systems, Oxford, United Kingdom), and viral load was measured by the Abbott m2000sp/rt (Uganda) or Roche COBAS Amplicor Monitor v1.5 (Zimbabwe) system. Total CD4^+^ and CD8^+^ T cells were measured in real time. In Uganda, children in the immunology substudy underwent whole-blood immunophenotyping, using anti-CD4-PerCP (Becton Dickinson [BD]), anti-CD45RA-APC (Caltag Medsytems), anti-CD31-PE (eBioscience), and either anti-Ki67-FITC (BD; after nuclear membrane permeabilization) or anti-HLA-DR-FITC (BD), with data acquired on a BD FACSCalibur flow cytometer. Analysis was undertaken using Cellquest (BD).

In the immunology substudy, viral loads were assayed at weeks 4, 24, 36, and 48 after ART initiation and every 24 weeks thereafter. Viral load response was defined as an HIV viral load of <10 000 copies/mL or a >1 log_10_ decrease in viral load at week 4, a viral load of <5000 copies/mL at week 24, and, subsequently, viral loads of <80 copies/mL at all measurements through 3 years (the lower limit of 80 copies/mL was selected because many samples had to be diluted owing to small volumes), allowing >80 copies/mL at week 36 only, provided that the viral load was exhibiting a decreasing trend (as observed among slow responders). Children were classified as having blips if the viral load returned to <80 copies/mL. Those in whom the viral did not return to <80 copies/mL but remained <5000 copies/mL were defined as having persistent low-level viral load, whereas individuals with a confirmed viral load of ≥5000 copies/mL after week 24 were defined as rebounders.

### Analysis

Analysis considered all inflammatory biomarkers, IL-7, age-associated CD4^+^ and CD8^+^ T-cell counts (hereafter termed “CD4 for age” and “CD8 for age,” respectively, and calculated as the ratio of the subject's CD4^+^ or CD8^+^ T-cell count to the count expected in healthy individuals of the same age; a maximum Spearman rho of 0.57 between any pair of parameters indicated a low risk of collinearity), and the following CD4^+^ T-cell subpopulations as a percentage of the total CD4^+^ T-cell population: CD45RA^+^ (naive), CD45RA^+^CD31^+^ (recent thymic emigrants), HLA-DR^+^ (activated), and Ki67^+^ (proliferating). These measurements were log_2_ transformed for normality; viral load was log_10_ transformed. Height for age and body mass index (BMI) for age were calculated using WHO reference values [20]; because weight for age does not cover the full age range, this was calculated using United Kingdom reference data [21]. To reduce the potential influence of outliers, measurements were truncated at the 2.5th and 97.5th percentiles (that is, values above the 97.5th percentile were set to the 97.5th percentile, and values below the 2.5th percentile were set to the 2.5th percentile).

As analyses included all substudy children and a sample of the remaining children, we prespecified an unmatched case-control design, using univariable rank sum and χ^2^ tests and multivariable logistic regression, forcing immunology substudy into the models as a stratifier. Sensitivity analyses used the Prentice method with time-to-event data for a case-cohort design [22], considering children from the immunology substudy as 1 subcohort and sampling 23% of cases who were not from the immunology substudy as a second subcohort. Variable selection was based on backward elimination with an exit *P* value of .05, including nonlinearity, based on fractional polynomial modeling, when *P* < .01 (Stata mfp). Interactions between variables in the final model were investigated and included when P_heterogeneity_ < .01.

To identify groups of children based on pre-ART laboratory parameters (CRP, TNF-α, IL-6, sCD14, and IL-7 levels; CD4 for age; CD8 for age; and viral load), we used principal components analysis (correlation matrix) followed by hierarchical clustering using complete linkage (with the number of clusters identified using the Calinski/Harabasz stopping rule). The independent interrelationships between laboratory parameters, age at ART initiation, pre-ART weight for age, pre-ART height for age, pre-ART BMI for age, and sex were identified using backward elimination (exit *P* value, .01; inclusion of nonlinearity when *P* < .001) from multivariable linear regression models for each laboratory factor in turn as the outcome and all other factors as explanatory variables. In children with immunophenotyping data, additional relationships between each factor and CD4^+^ T-cell subpopulations were identified (the proportion of recent thymic emigrant CD4^+^ T cells was not considered as it was strongly associated with the proportion of naive CD4^+^ T cells; Spearman correlation, 0.89); (exit *P* value, .05; inclusion of nonlinearity when *P* < .01, owing to smaller numbers).

All analyses were performed using Stata 14.1 (StataCorp). All *P* values are 2 sided.

## RESULTS

A total of 600 of the 1206 ARROW children were included by design; 115 were cases (54 died, 45 had new/recurrent WHO clinical stage 4 events, and 49 had poor immunological response; some children met multiple definitions), and 485 were controls. Among cases, deaths and WHO clinical stage 4 events occurred at a median of 19 weeks (range, 1–232 weeks) and 63 weeks (range, 1–212 weeks), respectively, after starting ART; in immunological non-response, the median CD4^+^ T-cell percentage over the first 3 years of ART was 7% (IQR, 3%–11%). Pre-ART biomarker data were available for 113 cases (98%) and 466 controls (96%); 1 control had a missing pre-ART viral load, leaving 578 children in the analyses. Immunophenotyping data were available for 170 controls (37%) and 9 cases (8%; Uganda only). In 299 children in the immunology substudy who had pre-ART biomarker and longitudinal viral load measurements, virological response was defined in 292 (98%) who were followed up at 24 weeks.

### Characteristics at ART Initiation

Cases were significantly older than controls (median age, 8.2 years [IQR, 4.4–11.4 years] vs 5.8 years [IQR, 2.3–9.3 years]) and had a lower pre-ART CD4^+^ T-cell percentage (4% [IQR, 1%–9%] vs 13% [IQR, 8%–18%]), a lower CD4 for age (median, 0.08 vs 0.29), and a lower ratio of CD4^+^ to CD8^+^ T cells (median, 0.1 vs 0.3; *P* < .0001 for all comparisons; Table 1). The ratio of CD4^+^ to CD8^+^ T cells was highly correlated with the CD4 for age (Spearman rho, 0.89; *P* < .0001). CRP, IL-6, and sCD14 levels were all significantly higher and the TNF-α level significantly lower in cases, compared with controls (*P* < .01). In multivariable logistic regression analysis considering all factors from Table 1, cases independently had a lower pre-ART CD4 for age (adjusted odds ratio [aOR], 0.56 per 2-fold increase [95% confidence interval {CI}, .49–.64]; *P* < .0001) and a higher pre-ART IL-6 level (aOR, 1.54 per 2-fold increase [95% CI, 1.18–2.01]; *P* = .002). There were no independent additional effects of pre-ART baseline CRP, sCD14, or TNF-α concentrations (*P* > .15), although a model containing CRP level instead of IL-6 level was similarly predictive (Akaike information criterion [AIC], 405 vs 404 in the original model), and a model containing CD4^+^ T-cell percentage instead of CD4 for age was only slightly less predictive (AIC, 412). There was a marginal trend toward cases independently having a higher pre-ART viral load (*P* = .08), a lower weight for age or BMI for age (*P* = .0503 or *P* = .08, respectively), and a lower CD4^+^ T-cell percentage (*P* = .06) than controls (other model coefficients were unchanged). Despite strong univariable effects, there was no independent effect of age (*P* = .23), ratio of CD4^+^ to CD8^+^ T cells (*P* = .22), WHO clinical stage (*P* = .48), or any other baseline factor (*P* > .1). Sensitivity analyses using Prentice time-to-event methods supported CD4 for age and IL-6 level as the most prognostic biomarkers (Supplementary Table 1).

**Table 1.** Characteristics at Antiretroviral Therapy (ART) Initiation and Impact on ART Response

Factor at ART Initiation	Overall (n = 578)	Cases (n = 113)	Controls (n = 465)	Univariable *P,* Cases vs Controls^a^	Multivariable OR (95% CI); *P* Value
Country/center				.89	
Uganda/Entebbe (nonurban)	81 (14.0)	18 (15.9)	63 (13.5)		…
Uganda/JCRC (urban)	146 (25.3)	28 (24.8)	118 (25.4)		…
Uganda/PIDC (urban)	150 (26.0)	27 (23.9)	123 (26.5)		…
Zimbabwe/Harare (urban)	201 (34.8)	40 (35.4)	161 (34.6)		…
In immunology substudy	299 (51.7)	16 (14.2)	283 (60.9)	<.0001	0.08 (.04–.15); <.0001
Male sex	287 (49.7)	62 (54.9)	225 (48.4)	.22	…
Age, y	6.3 (2.4, 9.7)	8.2 (4.4, 11.4)	5.8 (2.3, 9.3)	<.0001	…
Weight for age^b^	−2.3 (−3.4 to −1.4)	−2.7 (−4.2, −1.7)	−2.2 (−3.2, −1.3)	.0008	…
Height for age^c^	−2.5 (−3.4 to −1.6)	−2.4 (−3.5, −1.5)	−2.5 (−3.5, −1.6)	.89	…
BMI for age^c^	−0.7 (−1.6, 0.2)	−1.3 (−2.3, −0.5)	−0.6 (−1.5, 0.2)	<.0001	…
Viral load, copies/mL	225 600 (73 800–624 200)	275 100 (143 700, 663 800)	212 700 (62 900, 613 800)	.03	…
CD8^+^ T-cell percentage	51.0 (41.0, 60.0)	54.0 (46.0, 65.0)	50.0 (40.0, 60.0)	.0008	…
CD8 for age	1.9 (1.3, 2.8)	1.6 (1.0, 2.3)	2.0 (1.3, 2.8)	.0007	…
CRP level, mg/L	4.5 (1.4, 15.6)	7.8 (1.9, 23.3)	4.0 (1.3, 13.8)	.008	…
sCD14 level, mg/L	2.1 (1.7, 2.6)	2.4 (1.8, 2.9)	2.1 (1.7, 2.5)	.0007	…
IL-6 level, pg/mL	6.1 (4.7, 9.5)	8.1 (5.6, 11.6)	5.8 (4.6, 8.7)	<.0001	1.54 per 2-fold increase (1.18–2.01) ; .002
TNF-α level, pg/mL	22.6 (19.0, 28.0)	20.2 (17.5, 24.1)	23.3 (19.6, 28.6)	<.0001	…
IL-7 level, pg/mL	9.3 (3.8, 16.8)	8.8 (5.4, 17.6)	9.5 (3.5, 16.7)	.28	…
CD4^+^ T-cell percentage	12.0 (6.0, 17.0)	4.0 (1.0, 9.0)	13.0 (8.0, 18.0)	<.0001	…
CD4 for age	0.25 (0.11, 0.41)	0.08 (0.02, 0.19)	0.29 (0.17, 0.44)	<.0001	0.56 per 2-fold increase (.49–.64); <.0001
Ratio of CD4^+^ to CD8^+^ T cells	0.2 (0.1, 0.4)	0.1 (0.0, 0.2)	0.3 (0.2, 0.4)	<.0001	…
Hemoglobin level, g/dL	10.5 (9.5, 11.5)	10.5 (9.5, 11.4)	10.5 (9.5, 11.6)	.77	…
WHO clinical stage				.048	
1 or 2	172 (29.8)	25 (22.1)	147 (31.6)		…
3 or 4	406 (70.2)	88 (77.9)	318 (68.4)		…
CD4^+^ T-cell monitoring				.73	
Yes	295 (51.0)	56 (49.6)	239 (51.4)		…
No	283 (49.0)	57 (50.4)	226 (48.6)		…
ART strategy				.52	
Arm A (3TC, ABC, NNRTI throughout)	202 (34.9)	44 (38.9)	158 (34.0)		…
Arm B (ZDV for 36 wk)	190 (32.9)	37 (32.7)	153 (32.9)		…
Arm C (long-term ZDV, 3 NRTIs after wk 36)	186 (32.2)	32 (28.3)	154 (33.1)		…
Initial NNRTI				.46	
Nevirapine	356 (61.6)	73 (64.6)	283 (60.9)		…
Efavirenz	222 (38.4)	40 (35.4)	182 (39.1)		…
Primary caregiver				.0005	
Mother	317 (54.9)	45 (40.2)	272 (58.5)		…
Other	260 (45.1)	67 (59.8)	193 (41.5)		…
Missing^d^	1	1	0		…

Data are median values (interquartile ranges) or no. (%) of subjects. There was no evidence of interactions in the multivariable model (*P* > .1). See Supplementary Table 1 for sensitivity analysis using time to event rather than binary outcomes and the Prentice method to adjust for the case-control design.

Abbreviations: 3TC, lamivudine; ABC, abacavir; BMI, body mass index; CI, confidence interval; CRP, C-reactive protein; IL-6, interleukin 6; IL-7, interleukin 7; NNRTI, nonnucleoside reverse transcriptase inhibitor; NRTI, nucleoside reverse transcriptase inhibitor; OR, odds ratio; sCD14, soluble CD14; TNF-α, tumor necrosis factor α; WHO, World Health Organization; ZDV, zidovudine.

^a^ By rank sum or χ^2^ tests of observed data.

^b^ Because WHO reference values for weight for age only cover children <121 months, this variable was calculated using United Kingdom reference values, which cover the full age range of ARROW children (Spearman correlation between United Kingdom and WHO reference values, 0.99 for 451 children age <121 months).

^c^ Calculated using WHO reference values.

^d^ The mode was assumed in multivariate analyses.

### At ART Initiation, Children Fall Into Subgroups With Different ART Responses

**Figure 1.**
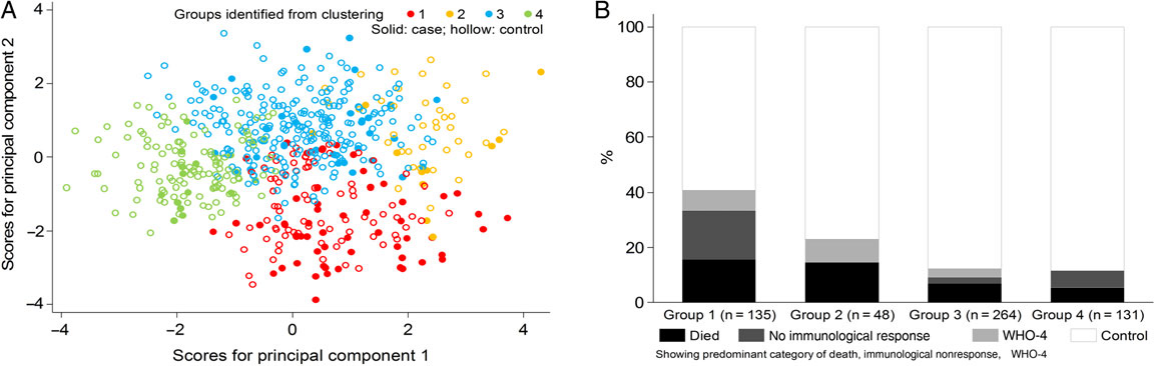
Subgroups of children at antiretroviral therapy (ART) initiation, identified from clustering of principal components. *A*, Compared to first 2 principal components. *B*, Compared to case vs control status. Abbreviation: WHO-4, World Health Organization clinical stage 4.

Whereas multivariable regression identifies the key determinants of poor outcomes, it does not inform how these risk factors are distributed across individuals. We therefore used principal components analysis to identify the most informative combinations of biomarkers, viral load, CD4 for age, and CD8 for age (Supplementary Table 2). Hierarchical clustering identified 4 groups of children at ART initiation, strongly associated with case versus control status (*P* < .001, by χ^2^ analysis; *P* < .0001, by logistic regression with adjustment for immunology substudy; Table 2 and Figure 1*A*).

**Table 2.** Subgroups of Children at Antiretroviral Therapy (ART) Initiation Identified From Clustering and Impact on ART Response

Variable	Group 1 (n = 135)	Group 2 (n = 48)	Group 3 (n = 264)	Group 4 (n = 131)	*P* Value^e^
Factors contributing to clustering					
CD4 for age	0.03 (0.01, 0.12)^a,b,c^	0.22 (0.12, 0.33)^b,c,d^	0.32 (0.22, 0.48)^a,d^	0.30 (0.21, 0.45)^a,d^	<.0001
Viral load, copies/mL	275 100 (145 100, 748 700)^c^	626 600 (144 900, 1254 900)^b,c^	272 000 (72 600, 673 700)^a,c^	107 200 (30 600, 285 300)^a,b,d^	<.0001
sCD14 level, mg/L	2.5 (2.0, 2.9)^b,c^	2.6 (2.2, 3.2)^b,c^	2.2 (1.6, 2.6)^a,c,d^	1.8 (1.4, 2.1)^a,b,d^	<.0001
CRP level, mg/L	6.4 (5.2, 9.2)^a,c^	35.3 (17.4, 76.3)^b,c,d^	4.5 (1.8, 12.1)^a,c^	1.2 (0.7, 2.8)^a,b,d^	<.0001
IL-6 level, pg/mL	6.4 (5.2, 9.2)^a,c^	26.4 (16.7, 40.0)^b,c,d^	6.7 (5.3, 9.4)^a,c^	4.2 (3.7, 5.0)^a,b,d^	<.0001
TNF-α level, pg/mL	19.8 (17.2, 23.5)^a,b^	27.8 (20.6, 33.0)^c,d^	25.8 (22.1, 31.7)^c,d^	19.1 (17.2, 21.9)^a,b^	<.0001
CD8 for age	1.2 (0.8, 1.6)^a,b,c^	2.2 (1.5, 2.7)^b,c,d^	2.4 (1.8, 3.6)^a,c,d^	1.7 (1.3, 2.4)^a,b,d^	<.0001
IL-7 level, pg/mL	11.0 (6.6, 19.0)^a,c^	6.4 (2.0, 16.9)^b,c,d^	11.3 (6.1, 18.1)^a,c^	2.9 (1.4, 7.0)^a,b,d^	<.0001
Baseline factors not contributing to clustering					
Age, y	7.9 (5.2, 11.0)	4.1 (1.7, 8.1)	3.6 (1.9, 8.1)	8.4 (5.1, 11.5)	<.0001
CD4^+^ T-cell percentage	3.0 (1.0, 8.0)	12.0 (5.5, 14.0)	13.0 (9.0, 18.0)	15.0 (11.0, 22.0)	<.0001
Hemoglobin level, g/dL	10.6 (9.4, 11.5)	9.6 (9.1, 10.6)	10.3 (9.3, 11.1)	11.6 (10.5, 12.1)	<.0001
Neutrophil count, ×10^9^ cells/L	1.8 (1.2, 2.6)	2.4 (1.5, 3.6)	2.3 (1.6, 3.1)	1.8 (1.5, 2.5)	<.0001
WHO clinical stage					.11
1 or 2	44 (32.6)	12 (25.0)	68 (25.8)	48 (36.6)	
3 or 4	91 (67.4)	36 (75.0)	196 (74.2)	83 (63.4)	
Current WHO clinical stage 3 or 4 illness at baseline^f^	42 (31.1)	21 (43.8)	111 (42.0)	19 (14.5)	<.0001
Tuberculosis at baseline	9 (6.7)	3 (6.3)	24 (9.1)	4 (3.1)	.17
Receiving antibiotic treatment at baseline (excluding tuberculosis treatment)	20 (14.8)	15 (31.3)	33 (12.5)	17 (13.0)	.008
Outcome					
Case	55 (40.7)	11 (22.9)	32 (12.1)	15 (11.5)	<.0001
Died	21 (15.6)	7 (14.6)	18 (6.8)	7 (5.3)	.03
Weeks from randomization to death	19.6 (12.4, 85.9)	5.1 (2.3, 15.3)	16.5 (6.7, 32.9)	136.9 (36.3, 172.0)	
WHO clinical stage 4 event	25 (18.5)	4 (8.3)	11 (4.2)	3 (2.3)	<.0001
Weeks from randomization to first WHO clinical stage 4 event	53.0 (6.4, 109.1)	104.1 (47.2, 117.6)	43.0 (26.0, 144.1)	156.4 (4.0, 161.9)	
Poor immunological response	31 (23.0)	0 (0.0)	8 (3.0)	10 (7.6)	<.0001
WHO clinical stage 3 or 4 or death	44 (32.6)	16 (33.3)	35 (13.3)	15 (11.5)	<.0001
Malnutrition as WHO clinical stage 3 or 4 or cause of death	10 (7.4)	4 (8.3)	8 (3.0)	2 (1.5)	.03
Tuberculosis	11 (8.1)	7 (14.6)	10 (3.8)	7 (5.3)	.02
Hospitalized	70 (51.9)	24 (50.0)	109 (41.3)	29 (22.1)	<.0001
Viral load response^g^					.20
Responded	20 (36.4)	5 (23.8)	36 (24.8)	29 (40.8)	
Had blip	21 (38.2)	8 (38.1)	73 (50.3)	24 (33.8)	
Had persistent low-level viral load	4 (7.3)	1 (4.8)	13 (9.0)	6 (8.5)	
Had rebound or no response	10 (18.2)	7 (33.3)	23 (15.9)	12 (16.9)	

Data are median values (interquartile ranges) or no. (%) of subjects.

Abbreviations: CRP, C-reactive protein; IL-6, interleukin 6; IL-7, interleukin 7; sCD14, soluble CD14; TNF-α, tumor necrosis factor α; WHO, World Health Organization.

^a^ *P* ≤ .05, by the rank sum test, compared with group 2.

^b^ *P* ≤ .05, by the rank sum test, compared with group 3.

^c^ *P* ≤ .05, by the rank sum test, compared with group 4.

^d^ *P* ≤ .05, by the rank sum test, compared with group 1.

^e^ Based on logistic regression analysis, accounting for immunology substudy as a stratifier for the case-control outcome; χ^2^ or rank sum tests were used otherwise.

^f^ See Supplementary Table 3 for details.

^g^ Defined only among 292 of 299 children in the immunology substudy who were alive and followed up at 24 weeks (5 died at ≤24 weeks; 2 died at 25–29 weeks without a viral load measurement after week 24). See “Methods” section for definitions. Only 2 children (one each in groups 3 and 4) were nonresponders, defined as never having a viral load of <5000 copies/mL.

Ordering by decreasing proportion of cases, group 1 (n = 135; 41% cases) was characterized by profound immunosuppression (median CD4^+^ T-cell percentage, 3.0%; *P* < .0001 vs other groups); group 2 (n = 48; 23% cases) was less immunosuppressed, and group 3 (n = 264; 12% cases) and group 4 (n = 131; 11% cases) were least immunosuppressed (*P* < .01 vs group 2). Despite their profound immunosuppression, the viral load in group 1 was similar to that in group 3 (*P* = .14) and only marginally lower than in group 2 (*P* = .07). Viral load was lower in group 4 than in all other groups (*P* < .0001). Median levels of the inflammatory markers CRP and IL-6 were highest in group 2 (35.3 mg/L and 26.4 pg/mL, respectively) and lowest in group 4 (*P* < .0001 for both markers vs all other groups). sCD14 level was significantly lower in group 3 than in both groups 1 and 2 (*P* < .0001) and lower in group 4 than in all other groups (*P* < .0001). TNF-α level and CD8 for age were significantly higher in groups 2 and 3 than in groups 4 and 1 (*P* < .05).

**Figure 2.**
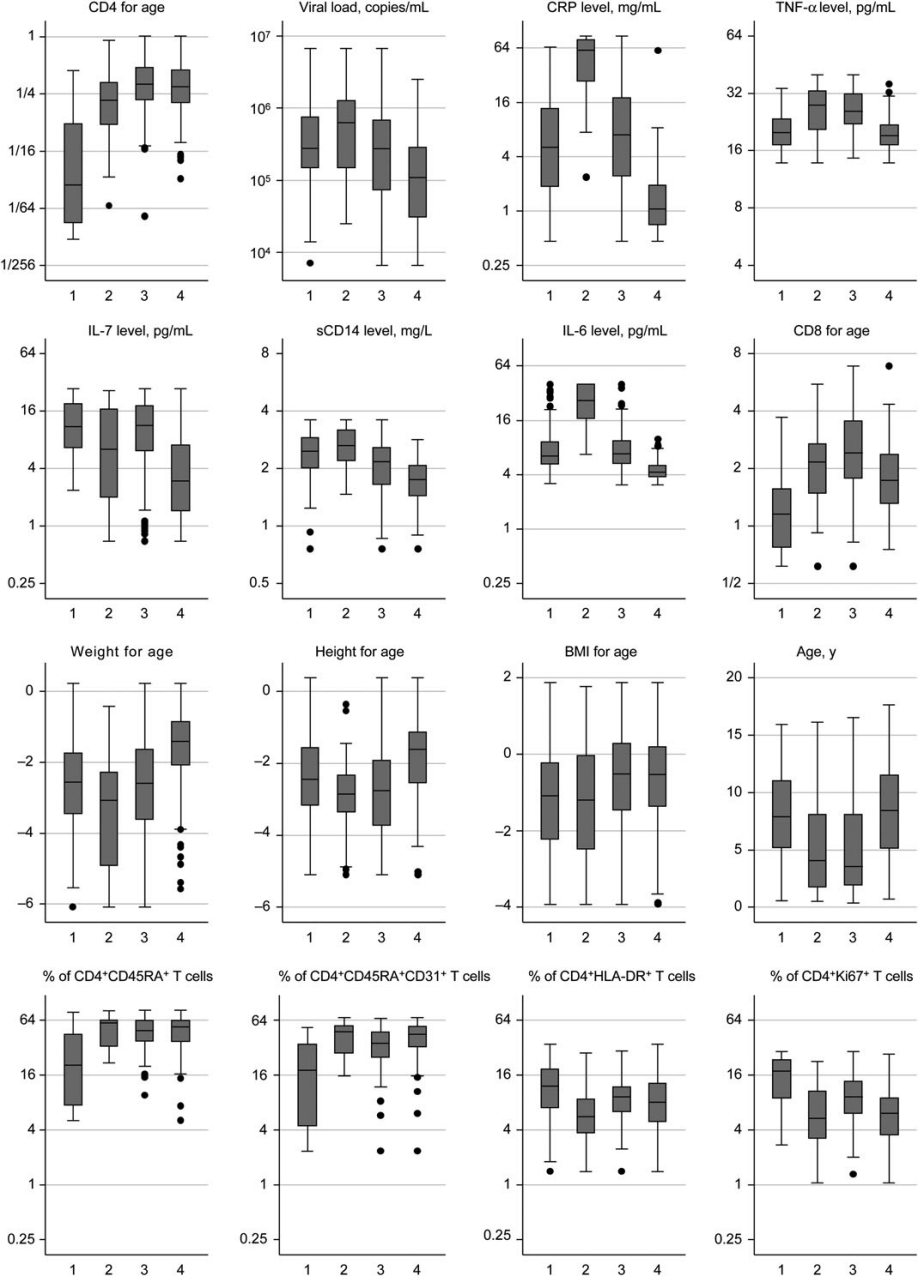
Characteristics in subgroups of children before antiretroviral therapy initiation. *P* < .0001 for all comparisons except the percentage of CD4^+^HLA-DR^+^ T cells (*P* = .03, by the rank sum test). CD4^+^ T-cell subpopulations (CD45RA^+^, CD45RA^+^CD31^+^, HLA-DR^+^, and Ki67^+^) measured in only 37, 13, 81, and 53 children in groups 1, 2, 3, and 4, respectively. The top 8 plots are factors used to define subgroups (through clustering of principal components). Abbreviations: BMI, body mass index; CRP, C-reactive protein; IL-6, interleukin 6; IL-7, interleukin 7; sCD14, soluble CD14; TNF-α, tumor necrosis factor α.

Considering factors not used to define subgroups, weight for age was highest in group 4 (*P* < .0001) and lowest in group 2 (*P* < .05; Figure 2). Height for age similarly was highest in group 4 (*P* < .0001) and lowest in groups 2 and 3 (*P* < .01). BMI for age generally increased across all groups. Whereas children in group 1 (median age, 7.9 years) were older than those in groups 2 and 3 (*P* < .0001), counterintuitively so were children in group 4 (median age, 8.4 years; *P* < .0001 vs groups 2 and 3). As expected, group 1 had the lowest proportion of naive (CD45RA^+^) and recent thymic emigrant (CD45RA^+^CD31^+^) CD4^+^ T cells (*P* < .01 vs other groups) and the highest proportion of activated (HLA-DR^+^) and proliferating (Ki67^+^) CD4^+^ T cells (*P* < .05). However, recent thymic emigrant proportions were also lower in group 3, compared with group 4 (*P* = .04), and the proportion of Ki67^+^ CD4^+^ T cells was higher in group 3 than in groups 2 and 4 (*P* < .05); there were no other significant differences in CD4^+^ T-cell subpopulations or activation markers among groups 2–4 (*P* > .05). Interestingly, group 2 also had lower pre-ART hemoglobin level, and group 2 (43.8%) and group 3 (42.0%) had more children with current WHO clinical stage 3 or 4 illnesses at baseline, compared with group 1 (31.1%) and group 4 (14.5%; *P* < .0001; Table 2 and Supplementary Table 3). Percentages of children with tuberculosis were similar in group 1 (6.7%; 9 subjects) and group 2 (6.3%; 2 subjects) at baseline; the greatest excess was in unexplained severe wasting/malnutrition (4 subjects [3.0%] in group 1 versus 10 [20.8%] in group 2). However, weight for age was only modestly negatively correlated with IL-6 level (Spearman rho, −0.28; *P* < .0001) and CRP level (Spearman rho, −0.15; *P* = .0002).

Taken together, group 1 comprised older children with profound immunosuppression, low proportions of naive and recent thymic emigrant CD4^+^ T cells, and high proportions of activated and proliferating CD4^+^ T cells; group 2 comprised younger children with less profound immunosuppression but high levels of inflammation and malnutrition; group 3 similarly comprised younger children with moderate immunosuppression, high TNF-α levels, and CD8 for age; whereas group 4 comprised older children with moderate immunosuppression, low viral loads, and low levels of inflammation. Groups 1 and 2 had similar overall mortality (16% and 15%, respectively), with deaths occurring shortly after ART initiation in both groups (median, 20 and 5 weeks), despite their significant differences in pre-ART CD4^+^ T-cell counts. The excess of cases in group 1 was predominantly due to immunological nonresponse (Figure 1*B*). The proportions with WHO clinical stage 3 or 4 events/death or hospitalization during ART generally decreased across groups 1–4, similar to the overall case proportion, although tuberculosis was more common in group 2 (14.6%) than in group 1 (8.1%; Supplementary Figure 2).

Virological responses following ART initiation were similar between groups; in particular, viral load suppression was not significantly poorer in group 1 (children with profound immunosuppression) than in other groups (*P* = .32, by the χ^2^ test). The main difference in virological response was a greater proportion of children with virological blips in group 3 (younger children with moderate immunosuppression but high TNF-α levels and CD8 for age, 50% of whom had had blips), compared with other groups (*P* = .02). There was also a trend toward more rebound/nonresponse in group 2 (high inflammatory markers; 33% with rebound or no response), compared with other groups (*P* = .054).

### Interrelationships Between Biomarker Levels, Viral Load, and Age at ART Initiation

Finally, we explicitly investigated the independent relationships between laboratory parameters and age at ART initiation in a cross-sectional analysis, considering each parameter in turn as the dependent variable (Supplementary Figure 3). CD4 for age was independently positively associated with CD8 for age and TNF-α level and negatively associated with IL-7 level, sCD14 level, viral load, and age at ART initiation; however, there was no independent association between IL-6 or CRP level and CD4 for age after adjustment for these other factors. IL-6 level was positively associated with viral load, CRP level, TNF-α level, and sCD14 level. Pre-ART CRP level was positively associated with only sCD14 and IL-6 levels, and sCD14 level was positively associated with only IL-6 and CRP levels and negatively associated with CD4 for age. Viral load was positively associated with TNF-α and IL-6 levels and negatively associated with CD4 for age and age. IL-7 level was negatively associated with CD4 for age and positively associated with TNF-α level but not with any other biomarkers. The only inflammatory biomarker independently associated with any cell subpopulation was TNF-α (positively with percentage of Ki67^+^ CD4^+^ T cells), although, as expected, the percentage of CD45RA^+^ CD4^+^ T cells was positively associated with CD4 for age, and the percentage of Ki67^+^ CD4^+^ T cells was negatively independently associated with CD4 for age.

## DISCUSSION

ART has transformed outcomes for HIV-infected children in sub-Saharan Africa [1]. However, pediatric treatment coverage is currently only 30%; therefore, large numbers of HIV-infected children will start ART over the next decade [2]. Historically, many children were older with advanced disease at ART initiation [23]; recent WHO guidelines recommend treatment for all HIV-infected children [24], so those starting ART might be expected to be younger, with less severe immunosuppression. However, this large cohort of African children shows that inflammation is a major pre-ART determinant of morbidity and mortality, independently of immunosuppression. Furthermore, a distinct group of younger children with high inflammation were at particularly high risk of early mortality during ART, despite only moderate immunosuppression, highlighting the need to target underlying pathogenic processes to improve outcomes in high-risk children, despite universal availability of ART.

Immunosuppression and immune activation are the hallmarks of HIV infection [25]. In the setting of chronic inflammation, there is increased CD4^+^ T-cell turnover and an impaired regenerative response to homeostatic signals [26, 27]. CD4^+^ T-cell depletion and inflammation are therefore highly interlinked processes [28] that drive disease progression in HIV-infected adults [9–11, 13, 15–17]. To our knowledge, ours is the first study to evaluate the associations between immunodeficiency, inflammatory biomarkers, and mortality in HIV-infected children, and it confirms that there are similarly 2 pathways (immunosuppression and inflammation) underlying serious clinical outcomes in pediatric HIV infection. IL-6 is the soluble inflammatory marker most strongly associated with mortality in most prior adult studies [9, 10, 13, 15, 16], and was the only biomarker to be associated with mortality independently of CD4^+^ T-cell count in our cohort. CRP was similarly predictive but did not have an independent additional effect in our models, consistent with their close biological interdependence, as IL-6 induces hepatic synthesis of this acute-phase protein [29]. We found no independent effect of TNF-α or sCD14 on morbidity and mortality. Viral load also had a less strong association with outcome than IL-6 or CD4^+^ T-cell count, and there was no independent effect of age or baseline disease stage. Whereas immune activation and inflammation are recognized to occur in HIV-infected children [30–42], the drivers of these processes are poorly characterized [18], and our analysis highlights the complex network of interrelationships between inflammatory markers (Supplementary Figure 3). The relative contributions of viral replication, coinfections, microbial translocation, malnutrition, and other factors across ages and populations may affect the precise inflammatory milieu and clinical outcome of an HIV-infected individual and may explain both the similarities and the differences between this study and prior adult studies [9–11, 13–17].

Using hierarchical clustering, we identified 4 subgroups of children with different patterns of pre-ART laboratory factors that were strongly associated with outcome during ART. Group 1 comprised children with low CD4^+^ T-cell counts and high clinical event rates, reflecting the well-recognized infection risk among children with profound immunosuppression [7]. This group also had the largest number of immunological nonresponders, likely because of failure of homeostatic CD4^+^ T-cell regenerative mechanisms. Group 2 had similar mortality to group 1 but comprised younger children with less severe immunosuppression but high levels of inflammation and malnutrition. This highlights the fact that the 2 important pathways to mortality identified in our study and others [16, 43, 44]—immunodeficiency and inflammation—do not necessarily overlap: some children have profound immunosuppression with modest inflammation, whereas others have moderate immunosuppression with profound inflammation. Most children in our cohort fell into groups 3 and 4: despite differences in age and viral load between these groups, pre-ART CD4^+^ T-cell counts were similar, and both groups had low event rates, reflecting the excellent clinical outcomes of many children initiating ART in sub-Saharan Africa [19].

These findings provide several insights into the pathogenesis of pediatric HIV infection and indicate potential approaches to identifying high-risk children and targeting interventions to improve outcomes. Where available, laboratory monitoring currently relies on CD4^+^ T-cell count and viral load to identify children at highest risk of early death. The CD4^+^ T-cell count has long been recognized as the best available prognostic marker, and, in this study, children with profound immunosuppression had high mortality, as expected. However, we also found that children with very similar pre-ART CD4^+^ T-cell counts can have very different clinical outcomes, depending on the prevailing inflammatory milieu. A point-of-care test measuring a soluble inflammatory biomarker such as IL-6 or CRP may identify children who are considered at high risk on the basis of their inflammatory status, rather than their immune status. These children may benefit from additional interventions at ART initiation to improve outcomes, although further studies are needed to characterize the drivers of inflammation in pediatric HIV infection, to inform what these interventions might be. Children in group 2 had higher rates of intercurrent infections and antibiotic use at baseline as compared to other groups, and overt infections may have partly driven pre-ART inflammation. These children also had high a high prevalence of moderate and severe wasting, and it is recognized that even asymptomatic malnourished children may have subclinical infections [45], which may have contributed to inflammation; we found a modest inverse association between IL-6 level and weight for age, providing some support for this premise. Furthermore, these children were more likely than those in group 1 to develop tuberculosis after ART initiation; although we did not have specific data on immune reconstitution inflammatory syndrome, it is plausible that some had subclinical tuberculosis at baseline that was unmasked after ART initiation. A pragmatic approach to reducing mortality may be to provide a package of interventions at ART initiation that are directed at multiple mechanistic pathways, including clinical and subclinical infections and malnutrition. The REALITY trial (ISRCTN43622374), currently underway in 4 countries in sub-Saharan Africa, is evaluating the independent and combined effect of additional antimicrobial, anti-HIV, and nutritional interventions at ART initiation on early mortality among adults and children with profound immunosuppression.

This study has several strengths and limitations. We studied a large, well-characterized cohort of children initiating ART across a range of ages and CD4^+^ T-cell counts in 2 high-burden countries, with longitudinal biospecimen collection and long-term outcome data. However, death and WHO clinical stage 4 events occurred in a relatively small proportion of children in the immunology substudy (the last 6 months of recruitment), who therefore formed more of the controls. All analyses adjusted for this factor, and analyses based on unmatched case-control or stratified case-cohort designs gave similar results, suggesting this had little influence, if any. Assessing WHO clinical stage 3 and 4 events can be difficult in children, and most diagnoses were presumptive. However, all reported WHO clinical stage 3 and 4 conditions (and deaths) were reviewed by an independent end-point review committee against diagnostic criteria prespecified in the trial protocol ensuring consistency. Since peripheral blood mononuclear cells were not available for the vast majority of children in this study, we were limited to measuring circulating plasma levels of cytokines and acute-phase proteins, rather than cellular responses following stimulation. Children were recruited in 2007–2008, at the start of pediatric ART rollout, and all met WHO criteria for ART initiation at the time and thus had moderate immunosuppression; further studies from different settings that include more children treated under universal ART guidelines, potentially with less severe immunosuppression, are needed to confirm our findings.

In summary, independently of immunosuppression, inflammation is a major driver of adverse outcomes during ART in HIV-infected children initiating ART in sub-Saharan Africa, similar to findings for adults [9–11, 13–17, 44]. Children most at risk of this inflammation-associated mortality have moderately preserved CD4^+^ T-cell counts and so will not be identified by current routine testing. Our data suggest that targeting HIV-related inflammation is a logical management approach in some high-risk children, as in adults. While most children initiating ART in sub-Saharan Africa have excellent long-term outcomes, a substantial minority die despite ART [3, 6, 8], and there is a critical need for novel approaches in this group, particularly as more children initiate ART under new universal treatment guidelines.

## Supplementary Data

Supplementary materials are available at http://jid.oxfordjournals.org. Consisting of data provided by the author to benefit the reader, the posted materials are not copyedited and are the sole responsibility of the author, so questions or comments should be addressed to the author.

Supplementary Data

## APPENDIX

### Study Team Members

#### Research Sites and Investigators

MRC/UVRI Uganda Research Unit on AIDS, Entebbe: P. Munderi, P. Nahirya-Ntege, R. Katuramu, J. Lutaakome, F. Nankya, G. Nabulime, I. Sekamatte, J. Kyarimpa, A. Ruberantwari, R. Sebukyu, G. Tushabe, D. Wangi, M. Musinguzi, M. Aber, L. Matama, D. Nakitto-Kesi, M. Kihembo, P. Pala, P. Kaleebu, S. Nassimbwa, and W. Senyonga; Joint Clinical Research Centre, Kampala, Uganda: P. Mugyenyi, V. Musiime, R. Keishanyu, V. D. Afayo, J. Bwomezi, J. Byaruhanga, P. Erimu, C. Karungi, H. Kizito, W. S. Namala, J. Namusanje, R, Nandugwa, T. K. Najjuko, E. Natukunda, M. Ndigendawani, S. O. Nsiyona, R. Kibenge, B. Bainomuhwezi, D. Sseremba, J. Tezikyabbiri, C. S. Tumusiime, A. Balaba, A. Mugumya, F. Nghania, D. Mwebesa, M. Mutumba, E. Bagurukira, F. Odongo, S. Mubokyi, M. Ssenyonga, M. Kasango, E. Lutalo, P. Oronon, G. Pimundu, L. Nakiire, E. D. Williams, O. Senfuma, L. Mugarura, J. Nkalubo, S. Abunyang, O. Denis, R. Lwalanda, I. Nankya, E. Ndashimye, E. Nabulime, and D. Mulima; University of Zimbabwe, Harare: K. J. Nathoo, M. F. Bwakura-Dangarembizi, F. Mapinge, E. Chidziva, T. Mhute, T. Vhembo, R. Mandidewa, M. Chipiti, R. Dzapasi, C. Katanda, D. Nyoni, G. C. Tinago, J. Bhiri, S. Mudzingwa, D. Muchabaiwa, M. Phiri, V. Masore, C. C. Marozva, S. J. Maturure, S. Tsikirayi, L. Munetsi, K. M. Rashirai, J. Steamer, R. Nhema, W. Bikwa, B. Tambawoga, E. Mufuka, M. Munjoma, K. Mataruka, Y. Zviuya, C. Berejena, and A. Shonshai; Zvitambo, Harare: P. Kurira and K. Mutasa; Baylor College of Medicine Children's Foundation Uganda, Mulago Hospital Uganda: A. Kekitiinwa, P. Musoke, S. Bakeera-Kitaka, R. Namuddu, P. Kasirye, A. Babirye, J. Asello, S. Nakalanzi, N. C. Ssemambo, J. Nakafeero, J. Tikabibamu, G. Musoba, J. Ssanyu, and M. Kisekka; and MRC Clinical Trials Unit at University College London, United Kingdom: D. M. Gibb, M. J. Thomason, A. S. Walker, A. D. Cook, A. J. Szubert, B. Naidoo-James, M. J. Spyer, C. Male, A. J. Glabay, L. K. Kendall, J. Crawley, and A. J. Prendergast.

#### Study Monitors and Committee Members

Independent ARROW trial monitors: I. Machingura and S. Ssenyonjo; Trial Steering Committee: I. Weller (chair), E. Luyirika, H. Lyall, E. Malianga, C. Mwansambo, M. Nyathi, F. Miiro, D. M. Gibb, A. Kekitiinwa, P. Mugyenyi, P. Munderi, K. J. Nathoo, and A. S. Walker, with the observers S. Kinn, M. McNeil, M. Roberts, and W. Snowden; Data Monitoring Committee: A. Breckenridge (chair), A. Pozniak, C. Hill, J. Matenga, and J. Tumwine; End-Point Review Committee (independent members): G. Tudor-Williams (chair), H. Barigye, H. A. Mujuru, and G. Ndeezi, with the observers S. Bakeera-Kitaka, M. F. Bwakura-Dangarembizi, J. Crawley, V. Musiime, P. Nahirya-Ntege, A. Prendergast, and M. Spyer; and Economics Group: P. Revill, T. Mabugu, F. Mirimo, S. Walker, and M. J. Sculpher.
