# Supplementary material for: Baseline Inflammatory Biomarkers Identify Subgroups of HIV-Infected African Children With Differing Responses to Antiretroviral Therapy
Source: J Infect Dis. 2016 May 18;214(2):226–36. doi: 10.1093/infdis/jiw148 (PMC4918830; doi:10.1093/infdis/jiw148)
Supplement: Supplementary Data [file supp_214_2_226__index.html]

Baseline Inflammatory Biomarkers Identify Subgroups of HIV-Infected African Children With Differing Responses to Antiretroviral Therapy — Baseline Inflammatory Biomarkers Identify Subgroups of HIV-Infected African Children With Differing Responses to Antiretroviral Therapy — Supplementary Data 

# Baseline Inflammatory Biomarkers Identify Subgroups of HIV-Infected African Children With Differing Responses to Antiretroviral Therapy

## Supplementary Data

Supplementary Data

- Supplementary Data - Docx file
