# Supplementary material for: Baseline Inflammatory Biomarkers Identify Subgroups of HIV-Infected African Children With Differing Responses to Antiretroviral Therapy
Source: J Infect Dis. 2016 May 18;214(2):226–36. doi: 10.1093/infdis/jiw148 (PMC4918830; doi:10.1093/infdis/jiw148)
Supplement: Supplementary Data [file supp_jiw148_jiw148supp.docx]

**Supplementary Table 1 Sensitivity multivariable analysis using the Prentice method**

|  | **Prentice method for case-cohort design** | | **Logistic regression including same factors (unmatched case-control)** | |
| --- | --- | --- | --- | --- |
| **Factor**  **(at ART initiation)** | **Multivariable hazard ratio (95% CI)** | **p** | **Multivariable odds ratio (95% CI)** | **p** |
| **Factors selected by Prentice method with p<0.1 in primary logistic regression** | | | | |
| In immunology substudy (vs no) | 0.12 [0.07,0.22] | <0.0001 | 0.08 [0.04,0.16] | <0.0001 |
| CD4 for-age (per 2-fold higher) | 0.63 [0.56,0.71] | <0.0001 | 0.56 [0.48,0.65] | <0.0001 |
| IL-6 (per 2-fold higher) | 1.53 [1.24,1.88] | 0.0002 | 1.52 [1.15,2.02] | 0.003 |
| BMI-for-age (per unit Z-score higher) | 0.78 [0.65,0.93] | 0.006 | 0.87 [0.71,1.07] | 0.19 |
| **Additional factors identified using the Prentice method** |  | | | |
| Country/Centre  Zimbabwe/Harare (urban)  Uganda/Entebbe (non-urban)  Uganda/JCRC (urban)  Uganda/PIDC (urban) | 1.00  2.80 [1.36,5.75]  1.04 [0.56,1.93]  1.59 [0.90,2.79] | 0.03  0.005  0.89  0.11 | 1.00  2.24 [.02,4.93]  0.98 [0.50,1.93]  1.24 [0.63,2.43] | 0.21  0.045  0.96  0.53 |
| Primary carer (mother vs other) | 0.43 [0.27,0.67] | 0.0002 | 0.72 [0.43,1.21] | 0.21 |

Note: no other factor in Table 1 provided additional (p≤0.05) prognostic information to the Prentice model. Although cases independently had lower pre-ART CD4-for-age, the magnitude (but not direction) of this effect varied by primary carer (weaker with mother; interaction p=0.0008) and centre (interaction p=0.007). In the logistic regression, case risk decreased with increased CD4-for-age regardless of BMI-for-age but, at the very lowest CD4-for-age (median ratio ≤ ~0.05), risk increased rather than decreased with increased BMI-for-age (interaction p=0.001). Other model coefficients similar. There were no additional (p≤0.01) interactions.

**Supplementary Table 2 Factor loadings from the principal component analysis**

|  | Principal component 1 | Principal component 2 | Principal component 3 | Principal component 4 |
| --- | --- | --- | --- | --- |
| % variation explained | 26% | 22% | 14% | 11% |
| CD4 for-age | -.25 | +.57 | -.27 | *+.08* |
| CD8-for-age | *-.09* | +.57 | *+.01* | +.15 |
| IL-7 (pg/mL) | +.26 | *-.08* | +.61 | +.66 |
| CRP (mg/L) | +.51 | *+.07* | -.41 | +.26 |
| IL-6 (pg/mL) | +.54 | *+.09* | -.27 | +.10 |
| sCD14 (mg/L) | +.40 | -.13 | -.19 | -.27 |
| TNFa (pg/mL) | +.22 | +.52 | +.26 | *-.06* |
| VL (log10 c/ml) | +.33 | +.19 | +.46 | -.62 |

Note: loadings <0.1 shown in italics and not described below

- Principal component 1 essentially represents a contrast between all the inflammatory biomarkers, IL-7 and VL (high levels imply poor status) vs CD4-for-age (high levels imply good status).
- Principal component 2 essentially represents a contrast between VL, TNF-α, CD4- and CD8- for age (response of the total CD4/CD8 compartment to virus and virus-associated cytokines) vs sCD14
- Principal component 3 essentially represents a contrast between CRP, IL-6, sCD14 (total inflammation) and CD4-for-age (CD4 response to inflammation) vs IL-7 (CD4 homeostasis), TNF-α (virus associated cytokines) and VL
- Principal component 4 essentially represents a contrast between CRP, IL-6, IL-7 and CD8-for-age vs sCD14 and viral load

**Supplementary Table 3 Current WHO 3/4 illness at baseline**

|  | Group-1 N=135  n (%) | Group-2 N=48  n (%) | Group-3 N=264  n (%) | Group-4 N=131  n (%) |
| --- | --- | --- | --- | --- |
| Current WHO 3/4 illness at baseline | 42 (31.1%) | 21 (43.8%) | 111 (42.0%) | 19 (14.5%) |
| Moderate unexplained malnutrition not adequately responding to standard therapy | 16 (11.9%) | 7 (14.6%) | 48 (18.2%) | 9 (6.9%) |
| Unexplained persistent diarrhoea | 2 (1.5%) | 1 (2.1%) | 6 (2.3%) | 0 |
| Unexplained persistent fever | 1 (0.7%) | 0 | 0 | 1 (0.8%) |
| Persistent oral candidiasis | 9 (6.7%) | 7 (14.6%) | 6 (2.3%) | 1 (0.8%) |
| Oral hairy leukoplakia | 1 (0.7%) | 0 | 1 (0.4%) | 0 |
| Pulmonary tuberculosis | 8 (5.9%) | 2 (4.2%) | 20 (7.6%) | 3 (2.3%) |
| Severe recurrent presumed bacterial pneumonia | 1 (0.7%) | 0 | 1 (0.4%) | 1 (0.8%) |
| Unexplained anaemia, neutropenia or thrombocytopenia for > 1 month | 0 | 0 | 1 (0.4%) | 0 |
| Chronic HIV-associated lung disease including bronchiectasis | 1 (0.7%) | 0 | 8 (3.0%) | 1 (0.8%) |
| Symptomatic Lymphoid interstitial pneumonia | 0 | 0 | 5 (1.9%) | 0 |
| TB lymphadenitis | 2 (1.5%) | 0 | 2 (0.8%) | 0 |
| Unexplained severe wasting or severe malnutrition not adequately responding to therapy | 4 (3.0%) | 10 (20.8%) | 15 (5.7%) | 3 (2.3%) |
| Pneumocystis pneumonia | 1 (0.7%) | 0 | 3 (1.1%) | 0 |
| Extrapulmonary TB | 1 (0.7%) | 1 (2.1%) | 3 (1.1%) | 1 (0.8%) |
| Oesophageal candidiasis | 3 (2.2%) | 0 | 1 (0.4%) | 0 |
| HIV encephalopathy | 3 (2.2%) | 1 (2.1%) | 16 (6.1%) | 2 (1.5%) |
| Details not known | 0 | 0 | 1 (0.4%) | 0 |

Events are not mutually exclusive therefore total to more than the total number of patients with WHO 3/4 illness at baseline


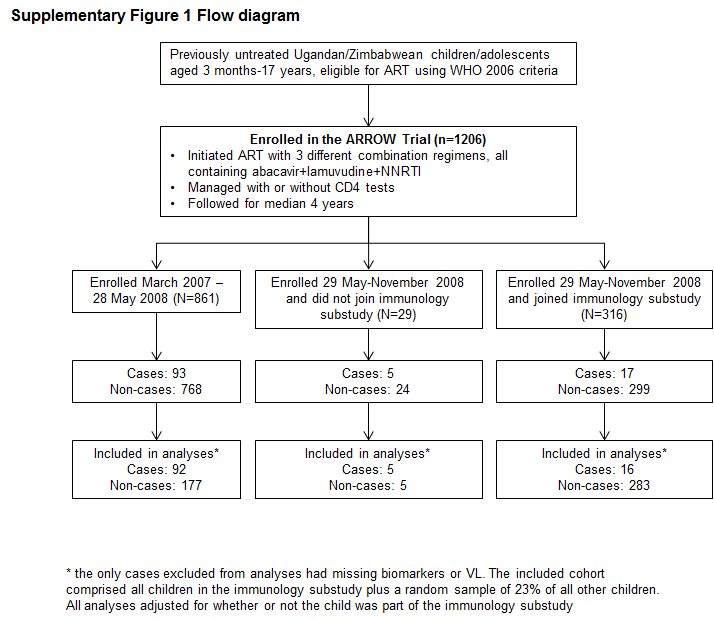


**Supplementary Figure 2 Impact of subgroup at ART initiation on subsequent TB**

**Supplementary Figure 3 Independent interrelationships between eight pre-ART laboratory parameters (CRP, TNF-α, IL-6, sCD14, IL-7, CD4-/CD8-for-age, VL), age at ART initiation and CD4 subpopulations**


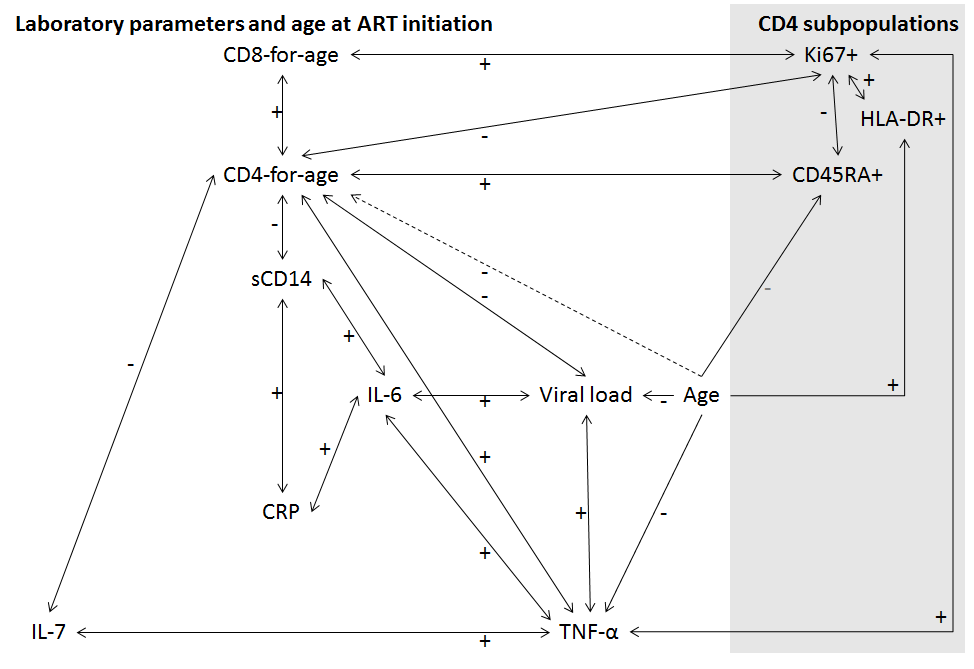


Note: each variable is modelled as a function of all other variables, using backwards elimination to identify independent predictors (see Methods). For example, CD4-for-age is independently predicted by TNF-α, sCD14, IL-7, CD8-for-age, VL, age at ART initiation (arrows to CD4-for-age; no independent effect of either IL-6 or CRP on CD4 after adjusting for these other factors). ”+” sign by the arrow indicates a positive relationship (e.g. high CD4-for-age associated with high CD8-for-age). “–“ sign by the arrow indicates an inverse relationship (e.g. high CD4-for-age associated with low IL-7). p<0.01 for all except the effects of sCD14 on IL-6 (p=0.014), TNF-α on IL-6 (p=0.06), TNF-α on Ki67+ (p=0.055) and CD45RA+ on Ki67+ (p=0.03). No independent effect of age on CD4-for-age after adjusting for CD45RA+ and Ki67+.
